# Experiences of NICU healthcare workers serving a high-risk population in a border community in Hidalgo County, Texas

**DOI:** 10.1038/s41372-025-02441-8

**Published:** 2025-10-07

**Authors:** Emily Gurwitz, Dynio Honrubia, Henry C. Lee

**Affiliations:** 1 School of Humanities and Sciences, Stanford University, Palo Alto, CA USA; 2 Women’s Hospital at Renaissance, DHR Health, Edinburg, TX USA; 3 Division of Neonatology, Department of Pediatrics, University of California San Diego, La Jolla, CA USA

**Keywords:** Health care, Health occupations

## Abstract

**Objective:**

Hidalgo County, Texas, located on the U.S.-Mexico border, is home to over 850 000 residents. Approximately 90% are Hispanic and 25% are foreign-born. Located here is the 65-bed level III neonatal intensive care unit at Doctor’s Hospital at Renaissance. This study aims to describe aspects of this unit that are unique and may have contributed to its success in serving a high-risk population.

**Study design:**

Semi-structured interviews were conducted with a variety of providers. The interviews were transcribed and qualitatively coded, and emergent themes were analyzed.

**Results:**

Thirty-four providers consisting of twenty nurses, three physicians, and eleven others participated. Emergent themes included the perceived importance of language and cultural concordance between patient families and providers and staff consistency.

**Conclusion:**

The non-clinical features of this unit reported on by its providers are deserving of additional research that clarifies their clinical effects and replicability at other institutions serving similar populations.

## Introduction

Disparities in NICU care on the basis of race and ethnicity, [1] language spoken by patient families, [2, 3] and socioeconomic status [4] have been characterized. Suggestions for advancing equity in care and outcomes include opportunities for improvement through both better technical and non-clinical care practices [5].

These disparities in healthcare may be of particular relevance in Texas’ Hidalgo County where over 90% of the population of 880,000 is Hispanic, 26% is foreign-born, 83% speak a language other than English in the home, and 33% of people under age 65 are uninsured [6]. Best estimates say that between 10 and 12% of the population is undocumented [7] and the county has a high incidence of maternal diabetes, hypertension, and obesity and inadequate prenatal care [8].

Located in Edinburg, Hidalgo County, Texas, just ten miles north of the U.S.-Mexico border, is Doctor’s Hospital at Renaissance’s (DHR) NICU. This 65-bed level III NICU was established in 2007 and admits about 1000 infants annually. Of those, approximately 130 are very low birth weight infants weighing less than 1500 grams. This NICU is notable because the mortality rate for infants born before 34 weeks’ gestation is 4.8%, substantially lower than national averages for infants born at the same gestational age (10.8% for non-Hispanic infants and 10.5% for Hispanic infants [9]). Therefore, DHR presents an opportunity to study an institution serving a low-income, non-White population with relative success.

We hypothesized that this NICU’s health care workers would be able to identify and share previously under-studied care practices and features of their NICU that might contribute to its success. Participants consisting of a variety of health care workers including nurses, physicians, and therapists reported on their impressions and experiences in semi-structured interviews. We aimed to gather in-depth qualitative data about these individuals’ perspectives and insights that can inform further research on equitable NICU care. This study’s fundings may be of particular relevance given the nation’s shifting demographics. The U.S. Hispanic population represents 19% of all Americans and the largest ethnic or racial minority group. Between 2022 and 2023, Hispanic individuals accounted for over 70% of all U.S. population growth [10]. NICUs across the nation stand to gain from the results of a study undertaken in this predominately Hispanic community.

## Methods

### Participants and setting

This qualitative study was approved by the institutional review boards at Stanford University and at DHR Health. A sample of 34 health care workers participated. Interviews took place in a private room adjacent to the NICU or in the private, in-hospital office of the participant.

### Procedures

Participants were recruited by a researcher in person in the NICU. We sought to interview a sample of health care workers representative of the unit in terms of gender, ethnicity, level of experience, and profession. This necessitated purposive sampling of some participants working in uncommon roles (i.e., mental health specialists, occupational therapists, and lactation consultants) and convenience sampling of others. Informed consent was obtained from all participants before participation.

A researcher conducted the interviews, which lasted between eight minutes and one hour and were audio recorded. The interviews were then de-identified and manually transcribed verbatim by a member of the research team. Individual interviews explored participants’ experiences working in DHR’s NICU and in other NICUs, if applicable. We used a semi-structured interview guide that covered a range of topics pertaining to the participant’s job responsibilities, impression of the clinical and non-clinical success of the unit, and evaluation of the development of the unit over time. Probing follow-up questions were asked when appropriate. The final section of the interview addressed the participant’s demographic information.

### Analysis

A sample of several selected transcripts and the interview guide were used to develop a preliminary codebook [11]. Because little is known about the experience of this group of health care providers, conventional content analysis was used to develop the initial codebook based on prominent themes and common responses [12]. The resulting codebook was revised and expanded as other common responses arose and until it required no modifications when applied to additional transcripts. Finally, codes capturing a large amount of data were analyzed for distinct subcategories, and the codebook was updated accordingly. Previously reviewed transcripts were re-coded according to the finalized codebook. For reliability, the research team met throughout the coding process to discuss changes to the codebook and emerging themes and subthemes.

## Results

### Participants

A sample of 34 of the unit’s health care workers consisting of twenty nurses, three physicians, two nurse practitioners, and nine others–including speech and occupational therapists and certified nursing assistants–participated. Twenty-six were female and eight were male. The participants ranged in age from 28 to 70. Twenty-two (65%) of those interviewed had worked in this NICU for more than ten years and several participants reported having worked in this unit since its inception. The participant with the least tenure had worked at DHR for three months. Eighteen (53%) of the participants reported being Hispanic.

All participants were employees of DHR’s NICU. Most worked full-time and exclusively in the NICU. One participant reported working part-time and two reported working both in the NICU and in other units of the hospital.

Despite the range of professions, years of experience, ages, and backgrounds of the participants, we identified four central themes from the data: workflow around interpretation; cultural concordance between patient families and staff; accepted and appropriate support for patient families; and staff consistency.

### Workflow around Interpretation

There was consensus among participants that a lack of Spanish fluency is a disadvantage in their work given that many patient families are exclusively Spanish-speaking (SS). There were reports about the availability of virtual interpretation services; however, no participants reported about having utilized this resource. When asked about translation, SS and non-Spanish-speaking (NSS) staff mentioned SS staff functioning as the unit’s interpreters. Since only three of seventeen self-reported SS staff are certified medical interpreters, most clinical staff members serve in an untrained capacity.

Many participants commented on the staff’s tendency to trade responsibilities while one communicates with another’s patient’s family in Spanish. This practice was most commonly reported by and about bedside nurses.

*“So, it’s kind of like, ‘Hey, come interpret with me and I can help you out.’ So, it’s kind of a two-way relationship. Can I do something for you while you interpret for me…?”*

Most comments about this informal system were positive. However, a few negative comments were made by SS participants about an increased workload and by NSS participants who expressed guilt about inconveniencing their colleagues even after offering them support with another responsibility.

Seven of the fifteen NSS participants reported some Spanish proficiency. Several described utilizing this knowledge to attempt to verify the accuracy and completeness of translation made by another provider. For example, one NSS participant remarked:

*“I can understand Spanish extremely well, so I know exactly [what is being said] when the nurse is telling the parent [about the patient].”*

### Cultural concordance between patient families and staff

**Fig. 1:**
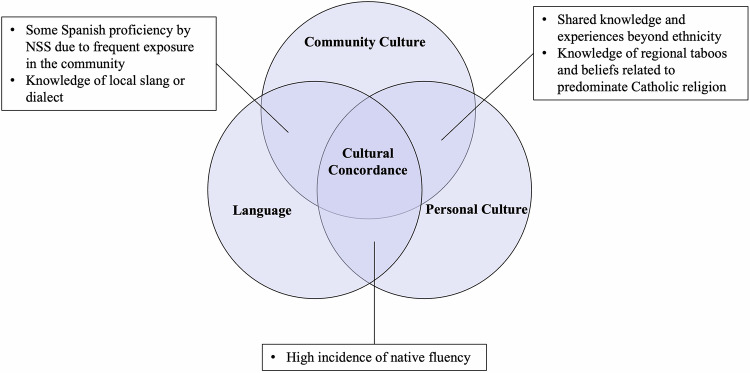
Cultural Concorance. Community culture, personal culture, and language as three distinct components of cultural concordance.

Discussion about communication with patient families uncovered the perceived importance of shared culture and how it only sometimes coincides with shared language (Fig. 1). Two main kinds of culture were mentioned. The first was personal culture, determined in part by a person’s ethnic, national, and religious background, and the second was local culture, created by the shared experience of living in this culturally distinct community. Nearly all participants reported sharing at least one kind of culture with most patients. Table 1 captures participants’ descriptions of how each of these distinct aspects of cultural concordance are apparent in practice.

**Table 1:** Selected quotations that highlight types of cultural concordance between patient families and NICU staff.

Speaker	Quotation	Notes
SS, non-local Hispanic provider	Even though I speak Spanish, I had to learn when I first moved here to adjust to their language even though it is Spanish. It’s different.	Spanish fluency alone is insufficient.
SS, Hispanic participant raised locally	Here in the Valley, we have a way of talking and we have slang or Tex-Mex, like it’s a combination of English and Spanish and made-up words in Spanish that nobody else uses, only the Valley.	Description of local knowledge that is distinct from general Spanish fluency.
SS, Hispanic participant raised locally	I would say a big 75%, 80% are uneducated, high-risk families. Low-income, with all those risk factors, and those parents, I feel that it’s very easy, they are very easygoing, and I think they are very easy for me to approach because of…we share the same culture.	Comments about relating to patients because of their socioeconomic status rather than traditional aspects of culture. Poverty and low educational attainment are common in the community.
NSS, non-Hispanic, non-local, participant who has lived in the Valley for > 5 years	Knowing the culture is really helpful. Staying in the Valley helps.	Implies that local culture can be learned.
Hispanic participant	Yes, and it’s easier because I am Hispanic. Sometimes I cannot say some things to an Anglo mom to break the ice. But [if] I have a Hispanic mom, there are some things that we do, like in every culture, that breaks the ice. The way we talk, the mannerisms, that break the ice.	Emphasizes the benefit of shared ethnicity.

Most participants have lived in the Rio Grande Valley for more than five years, given that 90% of participants have worked in this NICU for at least that long. Some non-locals commented on acquiring the local culture by living in the Rio Grande Valley for an extended period (Table 1). Though some cultural discordance relating to educational attainment was reported, the predominant theme was that NICU staff shares culture with patient families.

### Accepted and appropriate support for families

Local knowledge and intentional development of the NICU over time have led to the creation of several uniquely tailored non-medical supports for families that were mentioned frequently by participants, including a dedicated NICU mental health specialist, a full-time lactation consultant, and a no-cost follow-up clinic.

Participants commented about this NICU’s full-time, on-site mental health specialist who is a doctoral-level psychologist and a licensed professional counselor. Within five days of an infant’s admission to the unit, this specialist does an orientation with the family, which consists of psychoeducation and a tour of the unit. Then, about two weeks post-partum, mothers receive a mental health assessment that includes screening for anxiety and depression. Based on this, the specialist refers parents to the psychiatry department or suggests counseling. All parents and other caregivers are eligible for counseling for the duration of their infant’s stay in the NICU. This specialist also offers bereavement support and one-on-one education for the unit’s staff covering best practices for supporting patient families, their coworkers, and themselves.

Participants also reported about this unit’s dedicated, full-time lactation consultant. Participants report favorably on the variety of ways that this provides support to parents and note that the lactation consultant refers families to: Medicaid; the Women, Infants, and Children (WIC) Program; and the Rio Grande Valley Lactation Center for support in obtaining hospital-grade breast pumps and other supplies depending on their socioeconomic status and access to health insurance. Additionally, bedside nurses are educated annually on best practices related to interactions with patient families about breastfeeding.

Participants reported about this NICU’s no-cost, on-site follow-up clinic. Patients born weighing less than 950 grams and/or before 28 weeks’ gestation are referred to the clinic automatically and other patients are referred as needed on a case-by-case basis. Patients are followed by neonatology, speech and occupational therapy, and dietetics until two years of age. Participants commented on the importance of the follow-up clinic’s knowledge about and connection to local branches of public resources like WIC and Early Childhood Intervention. A nurse who works in the follow-up clinic reported about one common interaction with the local WIC office:

*“[The parents will] come back to me, and they’ll say, ‘Hey, WIC said that we can only get powdered [formula].’ And I’m like, ‘I know that is not the truth, because I have done it with other babies.’ So, sometimes it just needs specifying on the order: ready-to-feed only. And then I’ll contact WIC, and then I’ll talk to them.”*

### Staff consistency

65% (*n* = 22) of participants have worked in this NICU for over ten years. Of those, six have been with the unit since its inception. The unit has three neonatologists, all of whom have been with the unit since its inception. Participants reported positively about consistency in leadership, staff education, and personnel. On the other hand, participants reported negatively about the increased staff turnover rate caused by the Coronavirus pandemic. The most common negative experience reported about by participants was related to this phenomenon. Several commented on the difficulties of training new staff while being short-staffed and of losing experienced, tenured nurses who either retired or took other jobs during the pandemic.

Participants reported favorably about staff consistency created by dedicated staff members. The speech and occupational therapists, dietician, mental health specialist, lactation consultant, and respiratory therapists all work full-time and exclusively in the NICU. In response to a question about the benefits or limitations of working exclusively in the NICU, one therapist reported:

*“I think this is a blessing. Oh my gosh, to be able to stay in the unit and help. It’s not like I’m just coming in to see a couple of babies, because then you don’t get to develop relationships with the families, or even with the staff when you are just in and out. When you are here all the time, you see a lot more and you can do a lot more.”*

## Discussion

Our primary aim was to learn from health care workers about their experiences in DHR’s NICU. Through our semi-structured interviews and subsequent qualitative analysis, we uncovered four themes representative of the insights shared by the participants.

### Workflow around interpretation

Most participants report the ability to communicate with patient families in both English and Spanish. Bedside nurses described an informal system for communicating with SS families in which a NSS bedside nurse asks a SS provider to communicate with his or her patient’s parents directly while the NSS nurse takes over another responsibility for the SS provider. This workflow differs from conventional interpretation in many ways.

In most hospital settings, communication with SS patients and families is facilitated through professional medical interpretation services accessed on an as-needed basis. This service, provided via in-person interpreter, phone call, or videoconference, is typically external to the clinical care team, meaning that it might not provide the immediacy, continuity, or flexibility of communication needed in a NICU environment with many SS families. Additionally, dedicated interpreters conventionally serve as intermediaries between SS parents and NSS staff. This NICU’s informal system facilitates direct communication between SS parents and SS staff.

This informal system mitigates some challenges inherent to the conventional system of interpretation as SS staff in this NICU are always present and provide direct communication with SS parents and caregivers without an intermediary. Still, other problems, such as that providers tend to overestimate their Spanish fluency when they are asked to self-report [13], may persist. In this NICU, it is possible that this issue remains given that only some of the self-identified SS providers are certified medical interpreters.

Another aspect of this system worthy of further investigation is the consequences, if any, of the trading of responsibilities described by participants. It could be that the provider who temporarily assumes responsibility over another’s patient for the purpose of communication with that patient’s family may not be as familiar with the medical condition of that patient as the original NSS nurse. The effect of this complication on the quality of communication with SS families compared to English-speaking families remains unknown and warrants additional research.

Studies show that the infants of Spanish-speaking caregivers are at increased risk for several diseases common in the NICU, suggesting the importance of complete integration of Spanish-speaking families in the healthcare system [14]. By leveraging the availability of SS staff, this NICU aims to accommodate SS families. Additional evaluation of this system’s strengths and limitations can provide insight into the general effectiveness of this model of accommodation. Programs that reward SS providers directly for receipt of their formal interpreter certification and indirectly for their ongoing efforts as interpreters may support this workflow and alleviate negative feelings associated with SS providers’ increased responsibilities while ensuring high-quality communication with SS families. SS providers who opt not to participate in the certification process or whose language skills leave them ineligible for certification should not be discouraged from speaking to SS families in an informal context. Studies with adult patients find that professional interpretation successfully facilitates health education for limited English proficient patients; however, professional interpretation did not improve their impression of the quality of the interpersonal care they received [15, 16], suggesting that building rapport and increasing the frequency of communication with patients’ parents and caregivers by SS providers may be beneficial even when dedicated interpreters are involved in conversations regarding the patient’s medical status or prognosis.

In sum, with adequate incentives, formal certification requirements can support, rather than hinder, this model of accommodation involving direct communication between SS families and providers.

### Cultural concordance between patient families and staff

Another important theme is cultural concordance between patient families and providers. This study revealed the perceived importance of several distinct aspects of culture: community culture, personal culture, and language (Fig. 1). Each aspect can work alone or in combination to create a sense of shared experience and facilitate effective communication. For example, local knowledge contributes to cultural concordance between two locally born individuals even when they do not share the same ethnicity. Meanwhile, individuals who share ethnicity and language may be in cultural discordance if one does not know about local taboos, perspectives, and slang. Neither ethnicity, language nor local knowledge alone guarantees cultural similarity; experiences shared by participants illustrate how all three concepts can work together to create a sense of relatability and trust between patient families and providers.

Additional work should focus on whether having a mostly local workforce contributes to a feeling of shared experience and trust between parents and providers, as well as if this feeling of cultural concordance can be fostered in non-local, non-Hispanic, or NSS providers via cultural education, structured interactions with community members like former patients, or other programming. Whether these types of cultural concordance, perhaps through increased trust and communication and therefore increased caregiver preparedness for discharge or facilitated parent-infant interactions in the NICU, confer a measurable clinical benefit to patients is also deserving of additional study.

### Accepted and appropriate support for families

Participants perceived that the availability of comprehensive, culturally competent mental health care from a doctoral-level psychologist contributes significantly to this NICU’s success. While the majority of NICUs nationally provide some form of mental health screening for parents, most rely on social workers, nurses, or other non-psychologists to deliver these services [17]. Community-based NICUs are more likely than academic centers to lack even routine mental health screening for parents and caregivers [17]. In this context, DHR represents a relatively rare example of a community-based NICU offering in-unit, comprehensive mental health support from a doctoral-level psychologist, an approach that aligns with the National Perinatal Association’s recommendations for NICUs with 20 or more beds [18].

High-quality, easily accessible mental health care may be especially critical for this population given the elevated rates of postpartum depression (PPD) among Hispanic mothers [19], who are also among the least likely to access postpartum mental health care due to a variety of structural barriers [20]. Untreated PPD and other perinatal mental health conditions are known to negatively affect infant health and development [21]. The effect of a dedicated mental health specialist providing routine, on-site care to a predominantly Hispanic population of NICU parents and caregivers warrants further investigation in part because access to these providers is a modifiable and potentially replicable component of NICU care.

As with NICU mental health services, the presence of lactation consultants (LCs) and high-risk infant follow-up clinics for NICU families is generally considered standard practice. It is the specific structure and delivery of these services at DHR, which was emphasized by participants, that is notable. Fewer than half of NICUs nationwide employ a board-certified lactation consultant dedicated exclusively to the NICU [22], and little is known about the availability of language-concordant lactation support for SS mothers in non-academic settings. At DHR, participants reported that the SS LC and follow-up clinic staff assist families in accessing public health programs to obtain breast pump supplies and specialized formula. These efforts extend beyond the traditional scope of clinical care and may play a role in promoting good clinical outcomes for patients. Future research should aim to clarify this clinical effect, as the delivery of these supports represents a modifiable component of care that can be fine-tuned to serve other distinct populations.

### Staff consistency

Participants reported positively about the effects of physician and nurse longevity. It is known in other settings that increased nurse tenure leads to shorter lengths of stay [23]; however, this aspect of care remains under-studied in the NICU setting even as retention of nursing staff in a variety of settings remains a significant challenge [24, 25]. In this study, the majority of participants reported working in this NICU for more than ten years. Additional work should focus on what has facilitated staff longevity so that other NICUs with significant staff turnover can attempt to replicate these features. This work is especially warranted considering the increase in travel nursing and other changes to the nursing workforce during and after the Covid-19 pandemic [26].

### Study limitations

As with all qualitative research, the findings of this study are context-dependent and may not be generalizable to all NICU settings or patient populations. The data reflect the experiences and perceptions of participants within a single hospital that serves a predominantly Hispanic community in a medically underserved region. Certain features of this NICU such as the availability of Spanish-speaking staff and providers with a high degree of cultural concordance with patient families may not be present nor replicable in other institutions. Additionally, in settings with greater linguistic or cultural diversity among patients and families, even the aspects of care identified here as modifiable may be more difficult to implement or may require different approaches.

## Conclusions

This NICU serves a distinct population. Aspects of care reported on by its health care workers present opportunities for future research in other communities. Some of this unit’s unique features, including high levels of language and cultural concordance between providers and patient families and staff longevity may be the product of its mostly local workforce. Perhaps the prioritization of hiring locally born and/or locally educated nurses, physicians, and other staff can replicate these features in other unique communities. Additional research should aim to clarify the clinical effects of these non-clinical features.

## Data Availability

The datasets generated and analyzed in this study are not publicly available due to their sensitive nature. Data are available from the authors upon reasonable request for research collaboration.
